# Supplementary material for: Are carbon emissions trading and green financial instruments synergistic? -Comprehensive quantitative research based on content analysis
Source: PLoS One. 2024 Mar 7;19(3):e0298601. doi: 10.1371/journal.pone.0298601 (PMC10919601; doi:10.1371/journal.pone.0298601)
Supplement: S1 Data — (ZIP) [file pone.0298601.s001.zip › Data/地级市Z_F工作报告.zip!/║■▒▒/╦μ╓▌╩╨/└┤╫╘╣1⁄2╓┌║┼í╛┬φ┐╦╩2╛▌═°í┐_2014.docx]

各位代表：

现在，我代表市人民政府向大会报告政府工作，请予审议，并请各位政协委员和列席人员提出意见。

一、2013年工作回顾

刚刚过去的一年，面对宏观经济下行和持续干旱等严峻形势，我们在市委的坚强领导下，积极应对，竞进提质，大力实施“圣地车都”战略，深入开展“四大行动”，着力稳增长、促转型、惠民生、保稳定，全市经济社会继续保持又好又快的发展态势，主要经济社会指标均好于优于全省平均水平。初步核算，全年完成地区生产总值663亿元，较上年增长10.2%。预计完成规模以上工业增加值280亿元,同比增长13.5%；全社会固定资产投资658亿元,同比增长28%；地方公共财政预算收入29.67亿元，同比增长29.1%；城镇居民人均可支配收入和农民人均纯收入分别达到19733元、9387元，同比分别增长8.6%、11.5%。较好地完成了市三届人大三次会议确定的各项目标任务。

（一）经济发展稳中有进

——加快发展支柱产业，工业经济质效双增。围绕工业三年倍增计划，大力实施汽车机械、农产品加工“两大龙头”和冶金建材、医药化工、电子信息、战略性新兴产业“四个支柱”产业规划，六大产业发展迅猛，实现总产值970亿元，同比增长18%以上。“中国专汽之都”通过复审，2013中国（随州）专用汽车产业发展国际论坛成功举办，专用汽车及零部件、香菇、风机三个全省重点成长型产业集群通过考核。市场主体不断壮大，新登记2.3万多家，净增规模以上工业企业103家，总数达到632家。新认定高新技术企业15家以上，总数超过50家，提前两年完成“十二五”规划；高新技术产业实现增加值63亿元，占全市规模以上工业比重达到22.5%。新增省著名商标14件、省名牌产品9个。全年关停落后产能企业9家。

——成功抗击四年连旱，农村经济稳步增长。及时启动抗旱三级应急响应，全力以赴保饮水、保丰收、保稳定，最大限度降低灾害损失。农业结构不断优化，优质粮棉油、畜禽、瓜果菜、林木等产业板块发展壮大，粮食增产1亿斤，玉米等节水作物种植面积增长60%以上。农产品加工业产值与农业总产值之比达到2∶1，同比提高0.25个百分点。农产品出口创汇达到6.5亿美元，总额、增幅均居全省第一。农业产业化重点龙头企业新增26家，农民专业合作社新增167家。土地流转38.5万亩，占常用耕地面积的18%。农村基础设施建设得到加强，新建通村公路573公里，实施土地整治16万亩，完成病险水库除险加固251座，白云湖拦河闸除险加固工程、封江水库供水复线工程基本完成，鄂北地区水资源配置工程前期工作扎实推进，最严格水资源管理制度启动实施。

——积极扩大市场需求，第三产业持续向好。编制全市旅游产业发展规划，出台旅游业发展奖励办法，优化产业发展环境。成功举办癸巳年寻根节，首次在台湾联合举行“海峡两岸炎帝神农祭祀大典”，我市被授予“海峡两岸交流基地”。叶家山考古二期发掘收获重大成果，出土各类珍贵文物近2300件，中央电视台进行现场直播。随州大遗址保护片区规划建设被全国政协列入提案，安居遗址被列为第七批全国重点文物保护单位，擂鼓墩文物保护区内驻军搬迁取得实质性进展。旅游项目建设多点发力，大洪山核心景区建成开放，西游记漂流、中国千年银杏谷项目市场效应良好。全年接待游客1450万人次，旅游综合收入90.5亿元，同比分别增长20%、29%。消费市场规范发展，五眼桥市场被评为“全国诚信示范市场”。

全年实现社会消费品零售总额310亿元，同比增长13.5%，增幅位居全省前列。金融保险业加快发展，先后组织举办省金融机构支持随州发展政银企合作会议、“汉随直航”政银企恳谈会，建立小微企业金融服务基地，随州连续五年获授“湖北省金融信用市”，全年贷存比同比提高3个百分点。

——着力推进项目投资，发展后劲明显增强。深入开展“项目建设大行动”，亿元以上投资项目达到180个，是上年的1.8倍，组织实施市级重点项目178个，比上年增加70个。公路、铁路、供水、供电等基础设施建设扎实推进，麻竹高速随州西段、宁西铁路增建二线、新316国道、220千伏厉山变电站等项目进展顺利，城南新区路网建设全面启动，广水飞沙河供水工程建成运行。新能源项目建设迅速推进，二妹山和天河口风电场并网发电，武胜关等4个风电场开工建设，太阳能光伏发电“五个一百工程”全面启动，总投资60亿元的2×66万千瓦燃煤电厂成功纳入省规划。

（二）“神韵随州”建设发力

——城市规划建管得到加强。以“双百”城市为目标，编制城镇化与城镇发展战略规划、综合交通体系规划、历史文化名城保护规划等一批重大规划，制定城乡规划管理实施办法。重点实施中心城区“十大工程”，投资25亿元，完成城市道路、文化公园等21个市政项目。深入开展“洁美家园大行动”，实施“四治三化”、“门前三包”等综合整治，美化亮化街区立面，新建街头小游园8处、垃圾中转站9座、垃圾收集站点139个、公厕23座，启动“数字城管”项目建设，城市管理排名持续保持全省前列。城市品牌创建取得新成效，全国绿化模范城市、省环保模范城市成功创建，国家园林城市创建通过住建部实地验收，国家历史文化名城通过省级复查，省级卫生城市通过复查。

——城乡一体化扎实推进。广水市应广城区融合发展步伐加快，随县新县城建设取得新进展，市开发区平台建设加速，产业、人口聚集效应进一步显现。6个乡镇（办事处）跻身全省“百强乡镇”，杨寨镇成为全省“四化同步”试点乡镇，成功创建省级生态镇1个、生态村4个、宜居村庄19个，广水桃源村、曾都椅子山村纳入全国“美丽乡村”创建试点。全市城镇化率达到46%，同比增长2.78个百分点。

（三）改革开放实现突破

——改革力度不断加大。行政审批制度改革取得阶段性成效，422项市级审批事项减少为177项，其中80%以上的项目不予收费，审批时限比法定时限缩短68%。省可持续发展实验区正式获批创建，国家高新技术产业园区申报创建工作启动，省级文化旅游产业发展试验区申报创建取得积极进展。“营改增”扩围工作顺利推进，公务卡制度改革启动。金融市场体系加快健全，国有产权交易和企业股权托管机构成立，湖北银行随州分行即将挂牌开业，第二期15亿元城投债成功发行，泰晶公司上市申报文件被中国证监会正式受理。农村产权改革有序推进，全面完成村集体土地所有权确权登记发证工作。

——开放成效不断提高。全年外贸出口突破10亿美元，增幅居全省第一。实际利用内联资金156亿元、外资8770万美元，同比分别增长42%、25%。成功创建“国家级出口食用菌质量安全示范区”，中金黄金物流园、玉柴随州基地、航天汽车随州基地、大洪山整体开发等一批重大招商引资项目相继签约建设，随州大润发购物广场建成开业。积极争取各方面支持，与中国节能环保集团等一批央企和省直部门签署合作协议，赢得借力发展的更大空间。

（四）社会事业协调发展

以保障和改善民生为重点的 “十件实事”全面完成。财政保障民生支出74亿元，占公共财政预算支出的75%。扶持全民创业，增发小额担保贷款7000万元，城镇新增就业2.1万人，下岗失业人员再就业4000人，农村劳动力转移就业2.4万人，城镇居民登记失业率控制在4.6%以内。五项社会保险参保总数107.2万人次，城乡居民养老保险参保率99.2%，新农合参合率99.6%。启动城镇居民大病医疗保险和新农合大病商业保险，城镇居民、城镇职工、参合农民医保政策内住院费用综合报销比例分别达到71%、77%、78%。提高城乡居民最低生活保障标准和企业退休人员养老金待遇，发放各类救灾资金4800万元，保障困难群众正常生产生活。为5200名特困残疾人发放生活补贴，为500名残疾人免费配发辅助器具。新增廉租住房租赁补贴保障对象1410户，基本建成保障性住房、棚户区改造住房5062套，分配入住3626套。住房公积金扩面1.2万人，新增归集资金3.7亿元，当年使用3.2亿元。科技事业进步明显，我市荣获全国科技进步先进市。多渠道筹集资金2.6亿元，推进学校标准化建设，新建和改扩建幼儿园29所。发放扶贫资助金6000万元，惠及学生5.2万名。不断提升素质教育水平，实现义务教育均衡发展省级合格县（市、区）全覆盖。加快发展文体事业，市博物馆扩建、滨湖体育场主体工程竣工，城区社区文化活动室实现全覆盖，完成20户以下自然村广播电视“村村通”工程1175个。食品药品安全保障进一步加强。医疗卫生资源加快壮大，市中心医院新院建设进展顺利，市精神病医院、随县中医医院建成使用。低生育水平保持稳定，出生人口性别比综合治理效果明显。加大扶贫开发力度，减少贫困人口1.6万人。改造农村危房5400多户，完成清洁能源入户工程4400个。新建各类集中供水工程52处，农村新增受益人口24.1万人。城镇集中式饮用水源水质达标率100%，城区空气质量优于全省平均水平，环保四项约束性指标达到考核要求。

（五）精神文明、民主法治建设收获成果

推进各项文明创建活动，组织感动随州年度人物暨第三届道德模范、“随州好人”、“勤廉标兵”等评选表彰，开展“讲文明树新风”公益宣传，公民的思想道德素质和社会文明程度不断提升。推进法治建设，深化“六五”普法，市法律援助中心成功创建全国示范窗口，广水市、市地税局等21个单位荣获全国、全省法治创建先进单位。“三民”工程进入全面实施阶段，筹集社区民心公益基金近5000万元，推行社区网格化、“三自”物业和村务协理员管理服务模式。深入开展“平安建设大行动”，加强治安防控体系建设，城区设置监控摄像头1.2万个，建成“五警合一”巡逻防控网，每个社区组建专业治安巡逻队，“两抢两盗”案件同比下降30%，破获建市以来最大的摩托车系列盗窃案、盗卖文物案等，实现年度命案全破。认真开展安全生产大检查，安全生产态势总体平稳。加强信访维稳工作，畅通信访渠道，治理“三堵五闹”，打击网络谣言，社会大局和谐稳定。

（六）政府自身建设不断加强

坚持重大事项向市人大及其常委会报告、向市政协通报制度，自觉接受各方面的监督，全年办理人大议案建议、政协提案272件，满意率和基本满意率达到98%以上。加强政府管理创新，巩固“大部制”成果，推行部门“大科室制”，实施周报月评年考工作机制。深入开展“作风整顿大行动”，公开曝光处理一批“不在状态”干部，“三公”经费同比下降35%以上，“三短一简一俭”有效落实。扎实开展行风评议、电视问政活动，政风行风不断转变。推进“清廉随州”建设，我市反腐倡廉民意调查满意度在全省市州排名第二。

与此同时，新闻出版、文联、社科联、外事侨务、对台、民族宗教、对口援疆和国防教育动员工作继续加强，工会、青年、妇女儿童、红十字会、无偿献血、老龄和慈善事业加快推进，审计、统计、国调、国资监理、粮食、供销、机关事务、公共资源管理、档案、保密和地方志工作成效明显，邮政、通讯、无线电、石油石化、烟草专卖、人防、消防、水文、气象等工作取得新成绩。

各位代表！过去的一年，取得的成绩来之不易。这是省委、省政府正确领导的结果，是市委统筹全局、科学决策的结果，是市人大、市政协加强监督、参政议政的结果，是全市人民团结奋进、埋头苦干的结果。在此，我代表市政府，向全市人民，向所有纳税人，向各位人大代表、政协委员，向各民主党派、工商联、人民团体和无党派人士，向驻随部队、武警官兵和政法公安干警，向所有关心支持随州发展的同志们、朋友们，表示衷心的感谢，致以崇高的敬意！

在总结成绩的同时，我们也要清醒地认识到，我市经济社会发展还存在不少困难和问题：一是跨越赶超压力增大。受宏观经济下行和自身基础差、底子薄等因素影响，我市主要经济指标增速放缓，实现三年倍增的压力加大。二是产业实力依然较弱。农业抗御自然灾害和市场风险能力不强，工业产业层次不高，现代服务业发展不优，大项目、大企业缺乏。三是要素保障仍显不足。资金、人才、水资源等要素供应偏紧，一定程度上制约了发展。四是作风建设还需加强。少数政府部门和工作人员不作为、慢作为、乱作为等现象还不同程度存在，优化企业发展环境任重道远。对此，我们必须高度重视，采取过硬措施，认真加以解决。

二、2014年工作安排

2014年是全面贯彻落实党的十八届三中全会精神的开局之年，是深入实施“圣地车都”战略的重要一年。当前，我们面临的外部环境仍然复杂严峻，我国经济正处于换档期，下行压力依然较大，同时区域竞争更加激烈，给我市发展带来很多困难。但同时也要看到，国内经济长期向好的基本面没有改变，特别是党的十八届三中全会和中央经济工作会召开后，一系列全面深化改革的政策举措将出台实施，有利于形成新一轮经济增长周期，为我市加快发展带来重大机遇。只要我们坚定信心，迎难而上，抢抓机遇，创新发展，就一定能够在建设“圣地车都”的宏伟征程中开创发展新局面。

今年政府工作的指导思想是：认真贯彻党的十八大、十八届三中全会和中央、全省经济工作会议精神，按照“建成支点、走在前列”的总体部署和“五个湖北”建设的总体布局，以科学发展为主题，深入实施“圣地车都”战略，坚持“稳中求进、改革创新”的工作总基调，坚持“竞进提质、升级增效”的工作总要求，把改革创新贯穿于经济社会发展各个领域各个环节，加快转方式调结构，着力壮大特色产业，增强项目投资支撑，推进“神韵随州”建设，切实保障改善民生，促进经济社会持续健康发展，为全面建成小康随州奠定坚实基础。

今年全市经济社会发展的主要预期目标是：地区生产总值增长10.5%，规模工业增加值增长13.8%，全社会固定资产投资增长25%，地方公共财政预算收入增长17%，外贸出口增长11%，内联资金、实际利用外资分别增长20%和10%，社会消费品零售总额增长13%，城镇居民人均可支配收入增长10.8%，农民人均纯收入增长11%，居民消费价格涨幅控制在4%以内，人口自然增长率控制在6.5‰以内，单位生产总值能耗下降3%，全面完成节能减排等各项考核目标。

新的一年，将重点做好以下工作：

（一）推进产业转型升级，着力做大经济总量。围绕产业结构调整，巩固发展第一产业、做强第二产业、壮大第三产业，促进全市经济跨越赶超。

1、坚持工业兴市，提升竞争实力。深入实施工业三年倍增计划，着力构建特色鲜明、优势明显的现代工业体系。力争规模以上工业实现总产值1300亿元，同比增长20%以上。

调整优化主导产业。以“两大龙头、四个支柱”为主导，调整产业布局，优化产业结构，提升整体实力。突出抓好汽车机械产业，全力打造“中国专汽之都”。巩固提升现有基础,调整丰富产品结构，广泛应用新技术、新材料、新工艺，引导企业向“专精特新”转型，最大限度盘活存量。做强30公里专汽长廊，推动市开发区创建国家新型工业化专汽产业示范基地，加快建设品牌示范区。大力发展农产品加工业，改造提升冶金建材业，不断壮大医药化工业，着力推进电子信息业，积极培育战略性新兴产业。力争六大产业实现产值1200亿元，同比增长21%以上。

引导企业创新。加强技术创新，突出企业技术创新的主体地位，继续推动产学研合作，加大知识产权保护力度，促进一批科技成果产业化，抓好50个3000万元以上的重点技改项目，力争新增高新技术企业10家以上。加强产品创新，推进品牌强市建设，推动产品由制造向创造转变。加强管理创新，引导企业向内挖潜，强化成本、质量、资金管理，增强企业盈利能力和抵御风险能力，加快推进“三网融合”试点，启动4G网络前期建设，推进中小企业信息化建设，形成一批“两化”融合标杆企业。

培育市场主体。扶持壮大重点企业，以需求和问题为导向，深化“直通车”和“一企一策”服务，切实解决企业生产经营遇到的实际困难。引导生产要素向优势龙头企业聚集，培育行业领军企业，力争全年主营收入过20亿元的企业达到5家以上。实施中小企业成长工程，扶持小微企业发展，力争净增规模以上企业60家、小微企业1600家。加快发展混合所有制企业，通过引进大企业、大集团，推动关联重组、上下游联动重组、要素互补重组，让不同性质的资本交叉持股，实现企业借力发展。实施企业家素质提升工程，加强企业人才队伍培训。

加快发展县域经济和开发区经济。高度重视县域经济发展，支持随县打造全国重要的农产品出口基地和旅游强县，支持广水市建设中国风机名城，支持曾都区建成全国著名的铸造业聚集区。加快各级开发区建设，支持市开发区推进30平方公里“生态?产业新城”建设，推动市开发区与随县、广水、曾都开发区和十里工业园协同创建国家级高新区，着力提升园区的综合实力和核心竞争力。

2、稳固农业基础，放大特色优势。按照“农业要强、农村要美、农民要富”的目标要求，着力推进农业现代化。力争农业总产值实现240亿元，同比增长7%以上。

壮大特色农业规模。加强粮食安全工程建设，确保粮食产量稳定在160万吨以上。大力发展节水农业，扩大玉米、马铃薯等耐旱作物面积，着力发展泡泡青、菜豌豆、优势水产品等特色品种，加快发展食用菌规模化、标准化种植，推进资源转换和品种转型。大力发展木本油料产业，着力培植林业经济新的增长点。稳步推进“菜篮子”工程，加强“三品一标”认证工作，确保农产品质量安全。探索发展生态农业、旅游农业、高效农业，多渠道、多形式增加农民收入。

推进农业产业化进程。深入实施农产品加工业倍增计划，继续实施“四个一批”工程，力争全年农产品加工业产值达到500亿元，同比增长30%以上。培育壮大农业产业化重点企业，继续推动农产品加工企业向园区聚集，打造一批各具特色的农业产业化园区。

构建新型农业经营体系。坚持家庭经营在农业中的基础地位，推进集体经营、合作经营、企业经营等共同发展。因地制宜培育新型农业经营主体，新发展农民专业合作社100家以上，家庭农场50家以上。积极引导工商资本投入农业产业，向农业输入现代生产要素和经营模式。进一步探索农村产权制度改革。稳妥推进农民住房财产权抵押、担保、转让；加快随县整县推进土地确权试点工作，有序推进农村土地承包经营权流转，力争流转总面积达到40万亩以上，鼓励有条件的地方尝试开展农村土地承包经营权抵押贷款试点工作；规范森林资源资产流转行为。

改善农村生产生活条件。推进鄂北地区水资源配置工程，力争年底随州段顺利开工。加快实施河闸除险加固工程，完成100座病险水库除险加固任务。加快灌区改造与小型农田水利建设，推进灌渠畅通工程。加快中小河流治理，新建和加固堤防30.3公里。深入推进中低产田改造，继续实施土地连片整治。建成通村公路400公里，完成800个村广播电视“村村通”工程。开展专项扶贫、行业扶贫、社会扶贫，加快扶贫开发步伐。

3、突出文化旅游，繁荣第三产业。充分挖掘我市深厚的文化底蕴和富集的生态资源，打造 “世界华人谒祖圣地”，大力发展文化旅游业，积极发展商贸物流、金融保险等第三产业。

着力发展文化旅游业。加快省文化旅游产业发展试验区和随州大遗址保护片区创建工作，实质性推动“中国编钟音乐之都”建设，打造产业发展新载体。切实办好甲午年寻根节，力争取得更大成效。启动重点景区总体规划修编，全力推动炎帝故里风景区创建5A级景区，加快推进大洪山新区及慈恩寺配套建设，继续推进西游神话世界、千年银杏谷、三潭、桐柏山太白顶风景区等项目建设，力争创建4A级景区2个。加强旅游宣传营销，促进旅游资源整合，形成精品旅游线路，力争实现旅游综合收入97亿元，接待游客1560万人次。

大力发展商贸物流业。加快推进农产品现代流通体系和城市商贸综合体建设，培育大型综合批发市场和物流配送中心，继续开展“万村千乡市场工程”，积极发展农产品冷链物流，建设一批蔬菜直销店，兴建4处交通物流基础设施，形成多层次、多元化、多业态的商贸批零网络。促进电子商务应用，积极培育新的消费热点。

稳步发展金融保险业。推动社会融资总量持续扩大，新增信贷投放80亿元以上，贷存比提高3个百分点。加大“招行引资”力度，力争引进1家股份制商业银行，新设1家村镇银行，推进保险业健康发展，完善金融市场组织体系。

4、夯实底盘支撑，强化项目投资。坚持把投资和项目建设作为经济工作的主战场，不断夯实经济发展的底盘支撑。力争新开工亿元以上项目100个以上，竣工投产亿元以上项目100个以上，亿元以上项目完成投资达到全市投资总额的35%以上。

加大项目建设力度。围绕“投资增量提质、项目增效提速”目标，继续推进“项目建设大行动”，促进东风随州车轮厂整体搬迁等重大项目建设，支持恒天汽车工业园二期等重点项目尽早竣工，力争麻竹高速随州西段年底通车。

加大结构优化力度。认真研究国家产业政策，注重工业、能源等项目谋划，加强项目库建设，争取一批基础性、支撑性、功能性、公益性的重大项目，不断优化投资结构。积极促成武汉经随州至襄阳、十堰城际铁路年底前开工，扎实做好2×66万千瓦燃煤电厂和500千伏变电站等项目前期工作。

加大服务保障力度。坚持项目路线图推进制、项目建设现场观摩制、重点项目主官协调制和督办通报制等机制，健全领导挂点包保、企业缴费隔离带制度，全力抓好项目建设。坚持“一站式办公”、“一条龙服务”、“一个窗口对外”，推进 “绿色通道”、“并联审批”、“投资代办”、“一周办结”、建设项目收费“一表制”，完善投资环境评价考核机制，不断优化发展环境。切实抓好要素保障。积极争取中央、省专项资金和金融支持，推动发行10亿元建投债，开放投资领域，吸引民间投资，保障项目资金;充分利用职业教育优势，加强项目所需的专业人才培训;做好电网布局和电力设施建设，确保项目用电。

（二）推进城乡统筹发展，加快建设“神韵随州”。坚持发展以人为核心的新型城镇化，按照“一主两翼、多点支撑”发展格局，大力推动城镇规划上水平、城镇建设上速度、城镇管理上台阶。力争城镇化率提高2个百分点。

1、增强主城区辐射能力，着力建设“双百”城市

科学构架城市布局。启动城市总体规划修编，做好擂鼓墩文物保护区、城南新区等规划编制工作。坚持“东进、西优、南拓、北调”，进一步明晰中心城区功能分区定位，拓展优化城市空间布局，逐步打造城中商业居住综合区、城东高新技术产业区、城南行政金融文化会展区、城西文化旅游博览区、城北新型工业区和淅河城乡统筹示范区，形成功能互补、协调互动的城市空间格局。

加快建设城市设施。推进城市重点工程建设，续建编钟大道、新316国道、东护城河治理、青年路大桥、青年西路、市中心医院新院、省专汽研究院等14个重点工程，完成清河东路、明珠东路、城南片区道路等7条骨干路桥建设，启动南外环、随州传媒中心、擂鼓墩考古遗址公园、光华环保产业园（含城南污水处理厂）、编钟大道浪河至何店连接线等26个重大项目建设。

不断强化城市管理。继续推进城市品牌创建，适时启动国家卫生城市、国家双拥模范城等创建工作。创新城管执法体制，积极探索市区一体化“数字城管”新模式，力争入选全省智慧城市试点市。实行源头管控、综合治理、齐抓共管，切实加强和规范小区物业管理。全方位提升城市管理水平，力争城市管理始终走在全省第一方阵。

2、加快“两翼”发展，着力建设随县、广水城区

加快建设随县新县城，推进以县城为中心的交通、商贸、物流体系建设，不断完善行政新区和随县经济开发区的城市功能，创建生态园林县城。继续推进广水市应广两城同步发展，支持马都司生态新城、应山大道新区建设，完善道路交通设施和市政配套设施建设，打造新型中等城市。

3、构筑“多点”支撑，着力建设小城镇和农村新社区

编制实施县域村镇体系规划，扶持10个重点中心镇和10个特色镇建设。加快推进经济发达镇行政管理改革试点工作，积极推进杨寨镇全省“四化同步”试点镇建设，支持小城镇专业园区建设。扎实推进旅游名村、产业新村建设，重点建设15个精品村、30个示范村，着力打造30个宜居村庄。

（三）推进体制机制创新，深化改革扩大开放。坚持当前与长远相结合、局部与全局相配套、渐进与突破相衔接，着力优化体制机制，切实抓好各个领域各个环节的改革，全方位提升开放水平。

1、大力实施改革攻坚

进一步简政放权，深入推进行政审批制度改革，创新方式，优化流程，归并主体，缩短时限，提升行政效能。实施机构改革，坚持“大部制”，推行“大科室制”，切实抓好食品药品监督管理体制改革、卫生和计生部门整合、广播电视管理体制改革以及事业单位分类改革。着力推进财税金融创新。改进预算管理制度，实施全面规范的预算公开，强化财政预算执行绩效评价和政府投资项目预算评审，深入推进公务卡制度改革。不断深化投融资改革，加强政府融资平台建设，切实防控地方政府性债务风险。加大城投债券项目建设力度，做好中小企业私募债、集合债和集优债发行工作，努力破解企业贷款难“瓶颈”。全力推进多层次资本市场建设，力争企业上市取得实质性突破，推动企业在场外市场挂牌、股权托管和交易。加快要素市场化改革，稳妥推进公共资源交易市场建设，加快国有资产和涉农产权交易步伐。推进工商登记制度改革，逐步推行先照后证。探索户籍制度改革，推进农业转移人口市民化。

2、不断扩大对外开放

创新外贸发展方式。提高食用菌出口企业深加工水平，加快小龙虾、茶叶、蔬菜、中药材等特色农产品出口，打造湖北最大的农产品出口基地，力争农产品出口过7亿美元。促进专汽产业优势向出口优势转变，努力创建国家级汽车及零部件出口基地，逐步形成年出口过2亿美元的创汇能力。大力培育出口型新兴产业，建设年出口过1.2亿美元的通讯电子出口基地。把招商引资作为扩大开放的主攻方向，积极开展专业招商、产业链招商、园区招商和以商招商，提升招商引资的专业性、针对性、成功率，力争引进内联资金190亿元以上,实际利用外资9600万美元，引进固定资产投资额过亿元的项目40个以上。

（四）发展各项社会事业，努力改善民生质量。坚持民生导向，切实解决人民群众最关心、最直接、最现实的利益问题，确保人民安居乐业、社会安定有序。

1、全力抓好社会建设。协调发展社会事业，让发展成果更多更公平地惠及广大群众。

扎实推进教育发展。深化教育领域综合改革，不断提高教育质量。推进义务教育均衡发展，进一步改善办学条件，加强农村学校信息化建设，加快城区学校布局调整，缓解学校大班额问题。深化校企合作办学，提高职业院校主动服务地方经济社会发展的能力和水平。加强学校食品、校车等安全管理，确保教育平安稳定。

强化就业创业和社会保障。把稳定就业作为经济发展的优先目标，积极创造更多的就业岗位，统筹做好高校毕业生、农村转移劳动力、就业困难人员等重点群体就业工作。深入推进全民创业，完善创业政策、融资、孵化、培训、服务等平台建设，积极创建国家级创业型城市。加快建设覆盖城乡的社会保障体系，完善社会保险市级统筹机制，落实各项社会保险待遇。加强社会保障公共服务基础设施规范化建设，推进“金保工程”，尽早实现社会保障“一卡通”。加快建设城乡养老服务中心，促进农村福利院提档升级，扶持发展民办养老服务机构。关爱农村留守儿童、妇女、老人等特殊群体，做好城乡困难群众的帮扶救济工作。加快构建政府住房保障和市场供应相结合、满足多层次需要的住房供应体系，保持房价稳定。提高住房公积金缴存水平，继续推进非公有制企业扩面建立住房公积金制度。

发展文化医疗卫生等各项事业。加快市图书馆新馆等重点文体场馆建设，新建城乡体育健身工程100处，做好花鼓戏等非物质文化遗产的保护、开发与利用，落实文化惠民政策，提升公共文化服务质量。深化医疗卫生体制改革，扩大基本药物实施范围，做实公共卫生服务项目。加强医德医风建设，提高医疗服务水平。严格落实国家生育政策，加大“两非”案件查处力度。强化食品药品管理，着力整治餐饮等“五小”行业，积极创建省级食品安全示范市。加强对市场价格行为的监管，努力保持物价稳定。继续开展第三次全国经济普查。进一步做好其他社会事业。

加强和创新社会治理。继续完善基层社会治理和公共服务体系，做好社区、农村网格化和村务协理工作。按照“一社一品”要求，继续推进“三民”工程。有效激发社会组织活力，支持引导各类社会组织提供更为广泛和优质的公共服务。深化平安随州建设，打造人防、物防、技防工程，争创全省平安城市。持续开展安全隐患大排查，不断提升安全生产水平，高度重视信访维稳工作，切实稳定社会大局。

2、全力抓好生态文明建设。把生态文明建设放在更加突出的位置，融入经济、政治、文化、社会建设的各个方面。

深入开展“美丽乡村”建设。巩固深化“洁美家园大行动”，扎实开展新一轮“三万”活动，深入开展“美丽家园、清洁乡村”活动，积极推广桃源村、椅子山村全国“美丽乡村”创建试点村经验，加快实施市开发区“美丽乡村示范片”建设，有序推进农村环境综合整治，建设生态宜居家园。

继续实施绿化攻坚工程。再消灭宜林荒山20万亩，制定生态效益补偿政策，巩固完善首轮退耕还林成果，确保全市林地保有量不低于50.5万公顷。以创建全省林业生态示范县（市、区）为载体，大力推进城乡绿化一体化。

不断加强环境保护工作。开展生态文明示范区创建，加强饮用水源地环境保护和大气污染防治。强化环保标准硬约束，持续推进重点污染源排查整治、环境安全大检查等专项行动，严格执行主要污染物总量控制、环境影响评价、建设项目“三同时”等制度，进一步提升监管水平，保障全市环境安全。

努力打造资源节约型社会。重点抓好工业、交通、建筑、公共机构等领域的节能降耗。严格执行土地管理政策，进一步加大低效和闲置土地处置力度，推进国土资源节约集约利用。全面实施最严格水资源管理制度，建设节水型社会。积极申报创建国家新能源示范基地。

3、全力抓好精神文明和民主法治建设。深入推进城市文明、农村文明、行业文明和思想道德建设，倡导社会新风尚，广泛凝聚正能量。精心组织第四届道德模范和第五届“编钟文艺奖”等评选表彰活动，深入开展道德领域突出问题专项教育与治理，继续推进市民文明素质提升、城市环境面貌提升和文明程度指数提升三大工程。适时启动“法治城市”创建，深入开展“六五”普法，建立健全法律援助体系。强化行政执法责任制，进一步规范执法行为，大力推进文明执法、人性化执法。及时反映和协调人民群众的利益诉求，将涉法涉诉信访纳入法治轨道解决。

三、加强和改进政府自身建设

面对新形势、新任务，我们将进一步加强和改进政府自身建设，不断增强政府公信力和执行力，努力建设人民满意的政府。

（一）解放思想，紧扣“改革”大局。全面深化改革，政府首当其冲。各级政府和领导干部必须牢牢把握改革脉搏，打开思想解放的“总闸门”，冲破一切思想观念的束缚，消除一切体制机制的障碍,构筑投身改革的精神高地，营造支持改革的良好氛围，努力当好改革的倡导者。必须顾全改革大局，加快转变政府职能，正确处理政府与市场的关系，正确对待利益格局调整，以壮士断腕的非凡勇气，努力当好改革的推动者。必须更加注重改革的系统性、整体性、协同性，正确、准确、有序、协调地推进改革，胆子要大，步子要稳，努力当好改革的实践者。

（二）勇于担当，紧扣“责任”重心。当前，随州正处于爬坡过坎期，前进的路上有很多困难和挑战。各级政府和领导干部必须强化责任意识，发扬舍我其谁的担当精神，牢记发展使命，勇挑改革重担，敢啃硬骨头，敢攻硬堡垒，以实实在在的发展业绩向全市人民上交答卷。

（三）强化执行，紧扣“落实”关键。各级政府必须在党委的领导下，积极主动地开展工作，全面落实党委的各项部署和人大的各项决议，自觉加强同人民政协的民主协商，充分听取民主党派、工商联和无党派人士的意见建议，认真办理人大议案建议和政协提案，广泛吸纳社会各界的智慧与力量，不断提高科学决策、民主决策、依法决策水平。建立健全决策、执行、监督、考核、奖惩一体化的落实链条，形成雷厉风行、政令畅通的执行体系。严格目标管理，进一步完善周报月评年考工作机制，打造“乡镇大比武”等检验平台，加强绩效考核，提高工作成效。

（四）改进作风，紧扣“为民”根本。各级政府和广大干部必须坚决执行中央“八项规定”、省委“六条意见”和市委“实施细则”，扎实开展党的群众路线教育实践活动，全面加强作风建设，集中解决“四风”问题。健全领导干部带头改进作风、深入基层调查研究机制，完善直接联系和服务群众制度，切实问需于民、问计于民、问政于民，紧紧围绕群众最想的、最盼的、最愁的事情，真心实意为民排忧解难。深入推进“作风整顿大行动”、政风行风评议等活动，着力整治少数部门和干部“不在状态”问题，进一步提升政府形象。

（五）从严治政，紧扣“清廉”基石。各级政府必须以“清廉随州”建设为统揽，切实加强廉洁从政思想教育，深入推进惩治和预防腐败体系建设，健全严格的财务预算、核准和审计制度，厉行节约反对浪费，从严控制“三公”经费，构建“不能腐”的防范机制。严格落实党风廉政建设责任制和“一岗双责”制，规范执行领导干部报告个人有关事项的规定，深化政务公开，推进“阳光理政”，构建“不易腐”的保障机制。深入开展纠风治乱和专项治理，加强对公共服务行业和征地拆迁、农民负担等重点领域的监督检查，加大案件查处力度，始终保持惩治腐败的高压态势，构建“不敢腐”的惩戒机制。

今年市政府将继续办好“十件实事”：（1）扶持创业带动就业，增发小额担保贷款6000万元，扶持创业800人，城镇新增就业1.8万人。加大社会保险扩面征缴力度，基本实现各类群体全覆盖，按政策发放参保人员社保待遇。（2）加大教育投入，新建城南新区碧桂园学校和城东新区季梁学校，新建和改扩建幼儿园20所、农村初中校舍1.8万平方米，新建教师周转宿舍4000平方米。筹措资金4000万元，资助3万名贫困学生。（3）启动实施疾病应急救助制度，完善重大疾病保障制度，实现道路交通事故社会救助基金管理全覆盖。新建和改扩建村卫生室450个。继续免费对全市35—50周岁农村妇女进行“两癌”筛查，免费为1.2万对农村计划生育怀孕夫妇提供孕前优生健康检查。（4）加快养老服务体系建设，新建城市社区居家养老服务中心（站）100个、农村老年人互助照料活动中心100个。（5）继续实施保障性安居工程,建设公共租赁房800套，改造城市棚户区2100户。（6）深化平安随州建设。完善平安视频监控系统，设置各类探头1.3万个、管理平台49个，建设平安车场38个、平安车棚82个、平安停车点92个。（7）加强先觉庙、白云湖、封江水库等乡镇以上集中式饮用水源地污染防治，再解决14万农村人口安全饮水问题。（8）完成20个重点贫困村整村推进工作，减少农村贫困人口1.6万人。继续免费为筛查合格的0—6岁贫困残疾儿童实施抢救性康复，规范并扩面发放特困残疾人生活补助。（9）提升城市配套服务能力，在主城区新建压缩式垃圾中转站（点）8座，新建和改造特色小吃街3处，新建和改扩建一批农贸市场，建成“放心粮油”配送中心3个、连锁店45个。（10）全面完成主城区背街小巷硬化亮化任务。

各位代表！时代赋予重任，人民寄予厚望。让我们在市委的正确领导下，团结拼搏，改革创新，开拓进取，为深入实施“圣地车都”战略、全面建成小康社会而努力奋斗!
